# Clinical Significance of Hotspot Mutation Analysis of Urinary Cell-Free DNA in Urothelial Bladder Cancer

**DOI:** 10.3389/fonc.2020.00755

**Published:** 2020-05-19

**Authors:** Yujiro Hayashi, Kazutoshi Fujita, Kyosuke Matsuzaki, Marie-Lisa Eich, Eisuke Tomiyama, Makoto Matsushita, Yoko Koh, Kosuke Nakano, Cong Wang, Yu Ishizuya, Taigo Kato, Koji Hatano, Atsunari Kawashima, Takeshi Ujike, Motohide Uemura, Ryoichi Imamura, George J. Netto, Norio Nonomura

**Affiliations:** ^1^Department of Urology, Osaka University Graduate School of Medicine, Suita, Japan; ^2^Department Pathology, University of Alabama at Birmingham, Birmingham, AL, United States; ^3^Department Pathology, University Hospital Cologne, Cologne, Germany; ^4^Department of Therapeutic Urologic Oncology, Osaka University Graduate School of Medicine, Suita, Japan

**Keywords:** bladder cancer, *TERT* promoter, *FGFR3*, cell-free DNA, liquid biopsy, UroVysion, PCR, prognosis

## Abstract

Recent studies showed the clinical utility of next-generation sequencing of urinary cell-free DNA (cfDNA) from patients with urothelial bladder cancer (UBC). In this study, we aimed to develop urinary cfDNA analysis by droplet digital PCR (ddPCR) as a high-throughput and rapid assay for UBC detection and prognosis. We analyzed urinary cfDNA of 202 samples from 2 cohorts. Test cohort was designed for investigating clinical utility of urinary cfDNA, and was composed of 74 samples from patients with UBC, and 52 samples of benign hematuria patients. Validation cohort was designed for validation and assessment of clinical utility comparing urinary cfDNA with UroVysion (Abbott, Illinois, USA), and was composed of 40 samples from patients with UBC, and 36 prospectively collected samples from patients under surveillance after surgery for urothelial carcinoma. We performed ddPCR analysis of hotspot gene mutations (*TERT* promoter and *FGFR3*). In the test cohort, the sensitivity of urinary cfDNA diagnosis was 68.9% (51/74) and the specificity was 100% in patients with UBC. The sensitivity increased to 85.9% when used in conjunction with urine cytology. In addition, patients with high *TERT* C228T allele frequency (≥14%) had significantly worse prognosis in bladder tumor recurrence than patients with low *TERT* C228T allele frequency or negative *TERT* C228T (*p* = 0.0322). In the validation cohort, the sensitivity of urinary cfDNA was 57.5% (23/40) and the specificity was 100% in UBC patients. The sensitivity of the combination of urine cytology with our hotspot analysis (77.5%) was higher than that of urine cytology with UroVysion (68.9%). In the post-surgical surveillance group, patients positive for the *TERT* C228T mutation had significantly worse prognosis for bladder tumor recurrence than mutation negative patients (*p* < 0.001). In conclusion, ddPCR analysis of urinary cfDNA is a simple and promising assay for the clinical setting, surpassing UroVysion for detection and prognosis determination in UBC.

## Introduction

Urothelial bladder cancer (UBC) is one of the most common cancers in the world (1). Approximately 70% of UBC patients are diagnosed with non-muscle invasive bladder cancer at initial presentation (2). Non-muscle invasive bladder cancer is treated by transurethral resection of the bladder tumor (TURBT) and intravesical instillation therapy. Fifty to seventy percent of patients experience bladder tumor recurrence and 10–15% experience disease progression to muscle-invasive bladder cancer or distant metastasis (3, 4). For these reasons non-muscle invasive bladder cancer patients require cystoscopy, urine cytology, and computed tomography scans for a long period at regular intervals, but current follow-up methods are suboptimal due to their low sensitivity or high invasiveness (5).

Several urine-based diagnostic tools (UroVysion, NMP22, and etc) are approved for clinical use by The Food and Drug Administration. UroVysion is designed to detect aneuploidy for chromosome 3, 7, 17, and loss of 9p21 locus via fluorescence *in situ* hybridization (FISH) of cells in urinary sediments (6). Importantly, UroVysion can predict disease recurrence earlier than cystoscopy examination, suggesting that analysis of chromosomal changes by urinary analysis could predict disease recurrence in patients without visible tumors (7). However, none of these currently available tests are recommended for routine use due to their low sensitivity and high cost (2, 8).

There is an urgent need to develop useful and non-invasive assays for detection and surveillance of UBC. Several researchers have reported the utility of genomic analysis of urinary DNA from urothelial carcinoma patients by next-generation sequencing (NGS) (9–11).

NGS methods can produce a large amount of DNA data simultaneously, but a major hurdle in these methods is the data processing steps, or bioinformatics. Analysis by ddPCR can detect mutations with high sensitivity and is easy to interpret. In this study, we analyzed hotspot mutations in UBC using ddPCR analysis of urinary cfDNA focusing on *TERT* promoter and *FGFR3* mutations. Furthermore, we compared the clinical utility of cfDNA analysis with that of UroVysion.

## Materials and Methods

### Patients and Samples

To select genes suitable for ddPCR analysis, 66 UBC tissues of formalin-fixed paraffin-embedded (FFPE) samples from Japanese patients were obtained by transurethral resection of bladder tumor (TURBT) performed at Osaka University Hospital. The samples were then analyzed by massively parallel sequencing as previously described (Table S1) (12–16).

We analyzed 202 urine samples from 2 distinct and independent cohorts: test and validation cohorts. The test cohort consisted of 74 urine samples collected before TURBT (pre-TURBT group 1), and 52 samples collected from patients with microscopic or macroscopic hematuria with no malignant findings in the urinary tract, confirmed by a urologist (hematuria group). In this group, two patients with hematuria and positive urine cytology were also included as they revealed no malignant findings in the lower and upper urinary tract after a follow-up of more than 1 year, confirmed by a detailed examination by a urologist. All the samples of the validation cohort were collected prospectively to exclude the selection bias. The validation cohort was composed of 40 urine samples collected before TURBT (pre-TURBT group 2), and 36 samples collected from patients receiving the standard surveillance protocol after TURBT or radical nephroureterectomy with negative urine cytology (surveillance group) (Table 1). For the validation cohort, we performed both UroVysion assay and urinary cfDNA analysis. All patients were treated at Osaka University Hospital during 2013–2019 and provided written informed consent. This study was approved by the Institutional Review Board of Osaka University (IRB #668-3).

**Table 1:** Patients characteristics for urinary cell-free DNA analysis.

	**Test cohort**			**Validation cohort**		
	**Pre-TURBT group1 (*n* = 74)**	**Hematuria group (*n* = 52)**		**Pre-TURBT group2 (*n* = 40)**	**Surveillance group (*n* = 36)**	
	***n* (%)**	***n* (%)**	***p*-value**	***n* (%)**	***n* (%)**	***p*-value**
Age, median (range)	75 (31–91)	69 (38–89)	0.004	72.5 (47–89)	74 (44–89)	0.880
**Gender**						
Male	60 (81.1%)	38 (73.1%)	0.287	35 (87.5%)	31 (86.1%)	0.858
Female	14 (18.9%)	14 (26.9%)		5 (12.5%)	5 (13.9%)	
**Cytology**						
Positive	49 (66.2%)	2 (3.8%)	<0.0001	22 (55%)	0 (0%)	<0.0001
Negative	25 (33.8%)	50 (96.2%)		18 (45%)	36 (100%)	
**UroVysion**						
Positive	–	–		14 (35%)	3 (8.3%)	0.006
Negative	–	–		26 (65%)	32 (88.9%)	
Insufficient material	–	–		0 (0%)	1 (2.8%)	
**Prior therapy history before urine collection**						
Intravesical BCG therapy	3 (4.1%)	0 (0%)	0.142	5 (12.5%)	13 (36.1%)	0.016
Platinum-based systemic chemotherapy	1 (1.4%)	0 (0%)	0.400	2 (5.0%)	3 (8.3%)	0.558
**Pathological T stage**						
pTa	36 (48.6%)	–		21 (52.5%)	–	
pT1	23 (31.1%)	–		7 (17.5%)	–	
≥pT2	15 (20.3%)	–		8 (20%)	–	
No malignancy	0 (0%)	–		4 (10%)	–	
**Grade**						
High	56 (75.7%)	–		27 (67.5%)	–	
Low	17 (23%)	–		9 (22.5%)	–	
No malignancy	0 (0%)	–		4 (10%)	–	
Unknown	1 (1.4%)	–		0 (0%)	–	

*Urine samples of validation cohort were collected prospectively to exclude selection bias*.

### Pathological Diagnosis

Histological diagnosis was performed by experienced pathologists according to the 8th edition of the AJCC stage classification (17), and the World Health Organization 2016 criteria (18). The urine cytology was also evaluated by pathologists according to our institutional criteria. Positive urine cytology was defined to be class IV and V. We used the highest urine cytology class for data analysis of patients receiving several cytology tests.

### Sample Processing

The urine samples were processed to obtain the cfDNA following the method described in our earlier study (19). Briefly, post collection, the urine samples were centrifuged at 2,000 × g for 30 min, and the supernatants were stored at −80°C until use. Subsequently, the supernatants were thawed in a water bath at 27°C, and 12 mL of each supernatant was used for cfDNA purification after centrifugation at 16,000 × g for 10 min. The supernatant was processed by QIAamp Circulating Nucleic Acid Kit (QIAGEN, Hilden, Germany) as previously reported (19). The cell pellet was analyzed by UroVysion analysis.

### Massively Parallel Sequencing

Mutation and data analysis were performed by massively parallel sequencing that assign a unique identifier to each template molecule for increasing the sensitivity as previously reported. In brief, the *TERT*SeqS (12–16) assay which targets the promoter region of *TERT*, and the UroSeqS (12–16) assay which targets 10 genes (*CDKN2A, ERBB2, FGFR3, HRAS, KRAS, MET, MLL, PIK3CA, TP53*, and *VHL*) were performed on the 66 FFPE UBC tissue samples. Multiplex PCR was used to detect these mutations. The 59 primer pairs for this PCR were listed in Table S2. PCR products were purified AMPure XP beads (Beckman Coulter, PA, USA) and sequenced on an Illumina Miseq (Illumina, Inc., San Diego, CA, USA). The average unique coverage depth was 27,688 × (range 667–108,370 ×) for TERTSeqs and 3,356 × (range 51–21,650 ×) for UroSeqs. These data of Japanese patients were derived from the results published before (13, 14).

### Droplet Digital PCR (ddPCR)

Analysis of urine supernatants by ddPCR was performed on the QX100 Droplet Digital PCR System (Bio-Rad Laboratories, Hercules, CA, USA), including primers and probes (FAM, mutant type; HEX, wild type), and ddPCR Supermix for Probes (No dUTP) according to the manufacturer's protocol. Primers and probes (*TERT* promoters (g.1295228 C>T:C228T and g.1295250 C>T:C250T), and *FGFR3* S249C) for ddPCR, and PCR protocols were used as previously reported (19).

### Statistical Analysis

Statistical analysis was performed using JMP Pro 14.0.0 (SAS Institute Inc., Cary, NC, USA). The patient characteristics were compared using the Mann-Whitney U test and chi-square test. A log rank test was performed for analysis of the difference between the two groups. The Cox proportional hazard model was used for univariate and multivariate analysis. The best cutoff value was determined by receiver-operating characteristics curve analysis. Differences were considered statistically significant when the *p* < 0.05.

## Results

### Selection of Targeted Genes for Urinary cfDNA Analysis

At least one mutation was detected of 69.7% (46/66) of UBC tissues tested by massively parallel sequencing. *TERT* promoter mutations were detected in 48.5% (32/66) of tissues, TP53 mutations were found in 27.3% (18/66) of tissues, and *FGFR3* mutations were found in 24.2% (16/66) of tissues with high frequency. Although TP53 mutations are frequently found in UBC tissues, TP53 mutations are not suitable for ddPCR analysis because TP53 does not have hotspot mutations (Figure S1). However, *TERT* promoter mutations C228T and C250T, and *FGFR3* S249C are hotspot mutations with high frequency in UBC (Figure S1). We selected these 3 gene regions for urinary cfDNA hotspot mutation analysis by ddPCR.

### Urinary cfDNA Analysis of the Test Cohort

**Figure 1:**
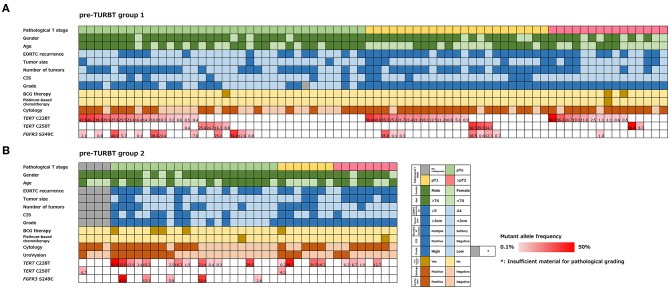
Alteration landscape of pre-TURBT group 1 **(A)** and 2 **(B)**, combined with tumor stage, gender, age, EORTC recurrence score, tumor size, concurrent CIS, grade, prior treatment history of BCG instillation and platinum-based chemotherapy, urine cytology, UroVysion, and mutant allele frequency (MAF) of each mutation.

**Figure 2:**
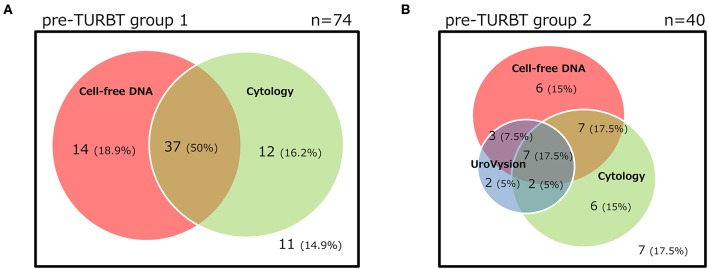
The distribution of positive results for each test of pre-TURBT group 1 **(A)** and 2 **(B)**.

**Figure 3:**
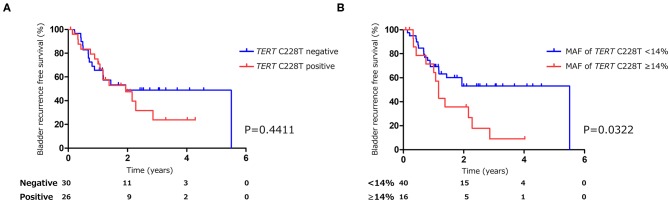
Kaplan-Meier analysis of bladder tumor recurrence free survival of pre-TURBT group1 stratified by *TERT* C228T mutation **(A)**, and by mutant allele frequency (14%) of *TERT* C228T **(B)**.

The positive rate of urine cytology was 66.2% (49/74) in pre-TURBT group 1, and 3.8% (2/52) in hematuria group (Table 1). Clinical-pathological features and mutant allele frequency (MAF) of each mutation are shown in Figure 1. Among the patients in the pre-TURBT group 1 before urine samples were collected, three (4.1%) patients had received at least one intravesical BCG therapy, and one (1.4%) patient had been treated with platinum-based regimens as adjuvant systemic chemotherapy for radical nephroureterectomy (Table 1, Figure 1A). The *TERT* C228T mutation was detected in both ≤ pT1 tumor and ≥pT2 tumor in pre-TURBT group 1 at high frequency (Table 2, Figure 1A). The *FGFR3* S249C mutation was mainly detected in ≤ pT1 tumor in pre-TURBT group 1 (Table 2, Figure 1A). There was no association between the positive rate of the three urinary cfDNA mutations and prior BCG instillation therapy history (*p* = 0.235) (Figure 1A). The sensitivity of urinary cfDNA analysis (*TERT* C228T, *TERT* C250T, and *FGFR3* S249C) was 68.9% in pre-TURBT 1, and the specificity was 96.2% using the hematuria group as the control cohort. When used in conjunction with urine cytology, the sensitivity increased to 85.1% in pre-TURBT group 1 (Figure 2A). Next, we analyzed the prognostic potential of urinary cfDNA. Although there was no association between presence of the *TERT* C228T mutation and bladder tumor recurrence, by stratifying the MAF of the *TERT* C228T at the best cut off point, the MAF of the *TERT* C228T mutation (≥14%) before TURBT was significantly associated with bladder tumor recurrence (*p* = 0.0322) (Figure 3). MAF of the *TERT* C228T mutation in urinary cfDNA before TURBT was an independent factor associated with bladder tumor recurrence after adjustment for European Organization for Research and Treatment of Cancer (EORTC) recurrence score and age (Table 3).

**Table 2:** Positive rate of urinary cell-free DNA, urine cytology, and UroVysion for pre-TURBT group 1,2 and hematuria group.

	**Pre-TURBT group 1**		**Hematuria group**	**Pre-TURBT gruop2**		
	**≤pT1 (*n* = 59)**	**≥pT2 (*n* = 15)**	**(*n* = 52)**	**≤pT1 (*n* = 28)**	**≥pT2 (*n* = 8)**	**No malignancy (*n* = 4)**
*TERT* C228T	28 (47.5%)	10 (66.7%)	0 (0%)	16 (57.1%)	4 (50%)	0 (0%)
*TERT* C250T	8 (13.6%)	2 (13.3%)	0 (0%)	1 (2.9%)	0 (0%)	1 (25%)
*FGFR3* S249C	19 (32.2%)	1 (6.7%)	0 (0%)	5 (17.9%)	0 (0%)	0 (0%)
Overall (*TERT* promoter and *FGFR3*)	39 (66.1%)	12 (80%)	0 (0%)	18 (64.3%)	4 (5%)	1 (25%)
Cytology	37 (62.7%)	12 (80%)	2 (3.8%)	14 (50%)	5 (62.5%)	3 (75%)
UroVysion	–	–	–	11 (39.3%)	2 (25%)	1 (25%)

**Table 3:** Univariate and multivariate analysis of factors associated with bladder tumor recurrence.

	**Univariate analysis**			**Multivariate analysis**		
	**Hazard ratio**	**95% CI**	***p*-value**	**Hazard ratio**	**95% CI**	***p*-value**
Age						
(≥75 vs. ≤ 74)	1.82	0.87–3.93	0.1118	2.81	1.25–6.59	0.0119
EORTC recurrence score						
(≥5 vs. ≤ 4)	2.44	1.14–5.65	0.0208	2.59	1.16–6.16	0.0193
*TERT* C228T mutation						
(≥10% vs. <10%)	2.16	1.03–4.52	0.0417	2.28	1.03–5.01	0.0410

### Urinary cfDNA Analysis of the Validation Cohort

**Figure 4:**
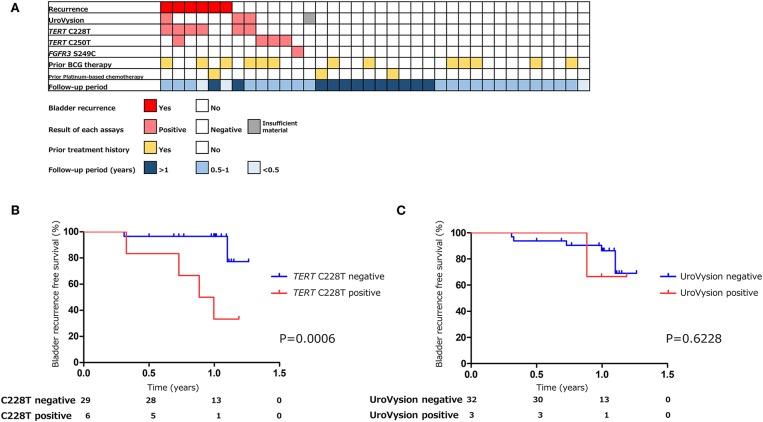
The association of urinary markers and bladder tumor recurrence in surveillance group (*n* = 36) **(A)**. Kaplan–Meier analysis of bladder tumor recurrence free survival of surveillance group stratified by *TERT* C228T mutation **(B)**, and UroVysion **(C)**.

The positive rate of urine cytology was 55% (22/40) in pre-TURBT group 2, and 0% in the surveillance group (Table 1). Among the patients in the pre-TURBT group 2 before urine samples were collected at the time of TURBT, five (12.5%) patients had received at least one intravesical BCG therapy, and two (5.0%) had been treated with platinum-based adjuvant chemotherapy for radical nephroureterectomy of upper tract urothelial carcinoma. Among the patients in the surveillance group, 13 (36.1%) patients had received at least one intravesical BCG therapy, and 3 (8.3%) had received platinum-based systemic chemotherapy before urine samples were collected (Table 1). Four patients were determined to have no evidence of tumor malignancy by pathological assessment of TURBT tissues in pre-TURBT group 2. Of these 4, one had the *TERT* C250T mutation, three had positive urine cytology, and 1 had a positive result by UroVysion (Table 2, Figure 1B). The positive rate of the 3 urinary cfDNA mutations was 57.5% in pre-TURBT group 2. There was no association between the positive rate of the three urinary cfDNA mutations and prior BCG instillation therapy or prior systemic chemotherapy in the pre-TURBT group 2 (*p* = 0.477), and surveillance group (*p* = 0.677) (Figure 1B). In addition, the sensitivity of urine cytology in conjunction with urinary cfDNA analysis (77.5%) was higher than when in conjunction with UroVysion (67.5%) (Figure 2B). Of the 36 patients in the surveillance group, 6 patients experienced bladder tumor recurrence during the follow-up period after sample collection (median: 364 days) (Figure 4). Of these 6 patients, 4 were positive for the *TERT* C228T mutation, but 1 patient tested positive by UroVysion assay. Patients in the surveillance group with the *TERT* C228T mutation had significantly worse prognosis for bladder tumor recurrence (*p* = 0.0006). This association was not detected by UroVysion assay (*p* = 0.6228) (Figure 4).

## Discussion

In this study, we showed the clinical utility of hotspot mutation analysis of urinary cell-free DNA by ddPCR for patients with UBC. This method aids in detection and surveillance of UBC in a non-invasive and simple manner. The sensitivity of urinary cfDNA analysis by ddPCR was high in both pre-TURBT groups. In combination with urine cytology, the sensitivity of urinary cfDNA assay was higher enough for clinical use than UroVysion. The mutant allele frequency of *TERT* C228T in urinary cfDNA before TURBT was significantly associated with bladder tumor recurrence. Furthermore, the utility of prognostic prediction of urinary cfDNA analysis of the TERT C228T was confirmed by samples prospectively collected in surveillance group. The *TERT* C228T mutation could predict disease recurrence more accurately than UroVysion.

Because urothelial carcinoma is directly and constantly in contact with urine, urinary cfDNA have clinical potential in cancer screening and disease monitoring of urothelial carcinoma (20, 21). There have been several studies on urinary cfDNA from UBC patients analyzed by NGS (9–11). Dudley et al. reported the usefulness of urinary cfDNA analysis by hybrid capture-based cancer personalized profiling by targeted deep sequencing (9). They reported that the sensitivity for early-stage bladder cancer was 84% (45/54), and urinary cfDNA mutations detected by NGS were significantly associated with bladder tumor recurrence (*p* < 0.0001). The sensitivity of urinary cfDNA analysis targeting *TERT* promoter and *FGFR3* mutations by ddPCR was lower than the NGS method, but the sensitivity was comparable to the NGS method when combined with urine cytology. Hotspot mutation analysis by ddPCR is simple enough for clinical use. We also showed that hotspot mutations of *TERT* promoter and *FGFR3* are frequently identified in tumor tissues of Japanese UBC patients consistent with those in previous report in Western countries (22). This confirms that our ddPCR method can be utilized for various ethnicities. Mutations in the upstream of *TERT* gene mainly affect two hotspots (C228T and C250T) (12, 23). This region recruits transcription factor and engages in long-range chromatin interactions (24, 25). The C228T mutation in the *TERT* promoter region has been shown to increase *TERT* expression more than the C250T mutation (26). We demonstrated that the *TERT* C228T mutation was significantly associated with bladder tumor recurrence in the surveillance group, and that ≥14% MAF of *TERT* C228T, and not just a positive for *TERT* C228T, was associated with bladder tumor recurrence in pre-TURBT group 1. This observation could be due to the volume of the *TERT* C228T reflect in bladder cancer activity, playing an important biological role in recurrence of UBC.

UroVysion is a FISH assay that detects aneuploidy of urothelial cells in urine. The overall sensitivity of UroVysion in previous studies varied from 36 to 86% (27, 28). This wide range of clinical performance might be due to bias in the selection of patients. In this study, the sensitivity of UroVysion in pre-TURBT group 2, in which samples were collected prospectively to exclude selection bias, was determined to be 35%. Kim et al. reported that UroVysion can predict disease recurrence and progression in patients with non-muscle invasive bladder cancer present as negative by cystoscopy (7). Of the 6 patients who experienced bladder tumor recurrence during the follow-up period, one had tested positive by UroVysion, and four patients tested positive for *TERT* C228T. *TERT* C228T analysis has been shown to predict disease recurrence more precisely UroVysion.

In this study, the MAF of urinary cfDNA was observed to be more than 50% in some cases (Figure 1), and as the occurrence of SNPs in the three gene regions evaluated in this study was rare (29), the mutated genes in urinary cfDNA were thought to be mainly derived from a urothelial tumor, not from normal cells.

This study has several limitations. First, the size of each patient group was small; especially, the number of patients who experienced bladder tumor recurrence was only six in the surveillance group. Larger size and multi-institutional study are needed to warrant current method. Second, the design of the primers needs cost-benefit optimization. The analysis of three gene regions by ddPCR presented in this study is useful in a clinical setting due to its simplicity. Addition of other mutated genes analyzed could increase the sensitivity but would increase the cost per test. Currently, our *TERT* promoter and *FGFR3* mutation analysis is sufficient for clinical use in combination with urine cytology. Finally, there could be possibilities of contamination by blood cells or epithelium though we performed centrifugation to remove cellular components in processing urine samples, which might result in a lower MAF than the actual value. Therefore, careful interpretation should be made for the cases with lower MAF in urinary cfDNA.

In conclusion, we demonstrated the clinical utility of *TERT* promoter and *FGFR3* hotspot mutation analysis in urinary cfDNA from UBC patients by ddPCR. The combination of urine cytology with urinary cfDNA analysis showed a higher sensitivity than when combined with UroVysion, enough to justify utilization of ddPCR analysis in the clinic. Liquid biopsy analysis of *TERT* promoter and *FGFR3* mutations in urinary cfDNA could be a novel diagnostic and prognostic biomarker for UBC.

## Data Availability Statement

The raw data supporting the conclusions of this article will be made available by the authors, without undue reservation, to any qualified researcher.

## Ethics Statement

The studies involving human participants were reviewed and approved by Osaka University. The patients/participants provided their written informed consent to participate in this study.

## Author Contributions

YH: conceptualization, formal analysis, methodology, investigation, and writing-original draft. KF: conceptualization, supervision, and writing-review and editing. KM: sample collection. M-LE: data analysis. ET: investigation and sample collection. MM, YK, KN, CW, and YI: investigation. TK, KH, AK, TU, MU, RI, and NN: supervision. GN: conceptualization and supervision.

## Conflict of Interest

GN equity or royalty from the licensed technologies from Johns Hopkins that are related to the work described in this paper. The remaining authors declare that the research was conducted in the absence of any commercial or financial relationships that could be construed as a potential conflict of interest.
