# Supplementary material for: Clinical Significance of Hotspot Mutation Analysis of Urinary Cell-Free DNA in Urothelial Bladder Cancer
Source: Front Oncol. 2020 May 19;10:755. doi: 10.3389/fonc.2020.00755 (PMC7250242; doi:10.3389/fonc.2020.00755)
Supplement: Supplementary file 1 [file Table_1.DOCX]

| **Discovery cohort (n=66)** | | |
| --- | --- | --- |
|  | **n** | **(%)** |
| **Age, median(range)** | **74** | **(33-87)** |
| **Gender** |  |  |
| **Male** | **53** | **(80.3%)** |
| **Female** | **13** | **(19.7%)** |
| **Pathological T stage** |  |  |
| **pTa** | **29** | **(43.9%)** |
| **pT1** | **25** | **(37.9%)** |
| **≥ pT2** | **12** | **(18.2%)** |
| **Grade** |  |  |
| **High** | **43** | **(65.2%)** |
| **Low** | **15** | **(22.7%)** |
| **Unknown** | **8** | **(12.1%)** |

Table S1

Discovery cohort of urothelial bladder cancer patients for selection of candidate gene mutations.
