# Supplementary figures and images for: Clinical Significance of Hotspot Mutation Analysis of Urinary Cell-Free DNA in Urothelial Bladder Cancer
Source: Front Oncol. 2020 May 19;10:755. doi: 10.3389/fonc.2020.00755 (PMC7250242; doi:10.3389/fonc.2020.00755)

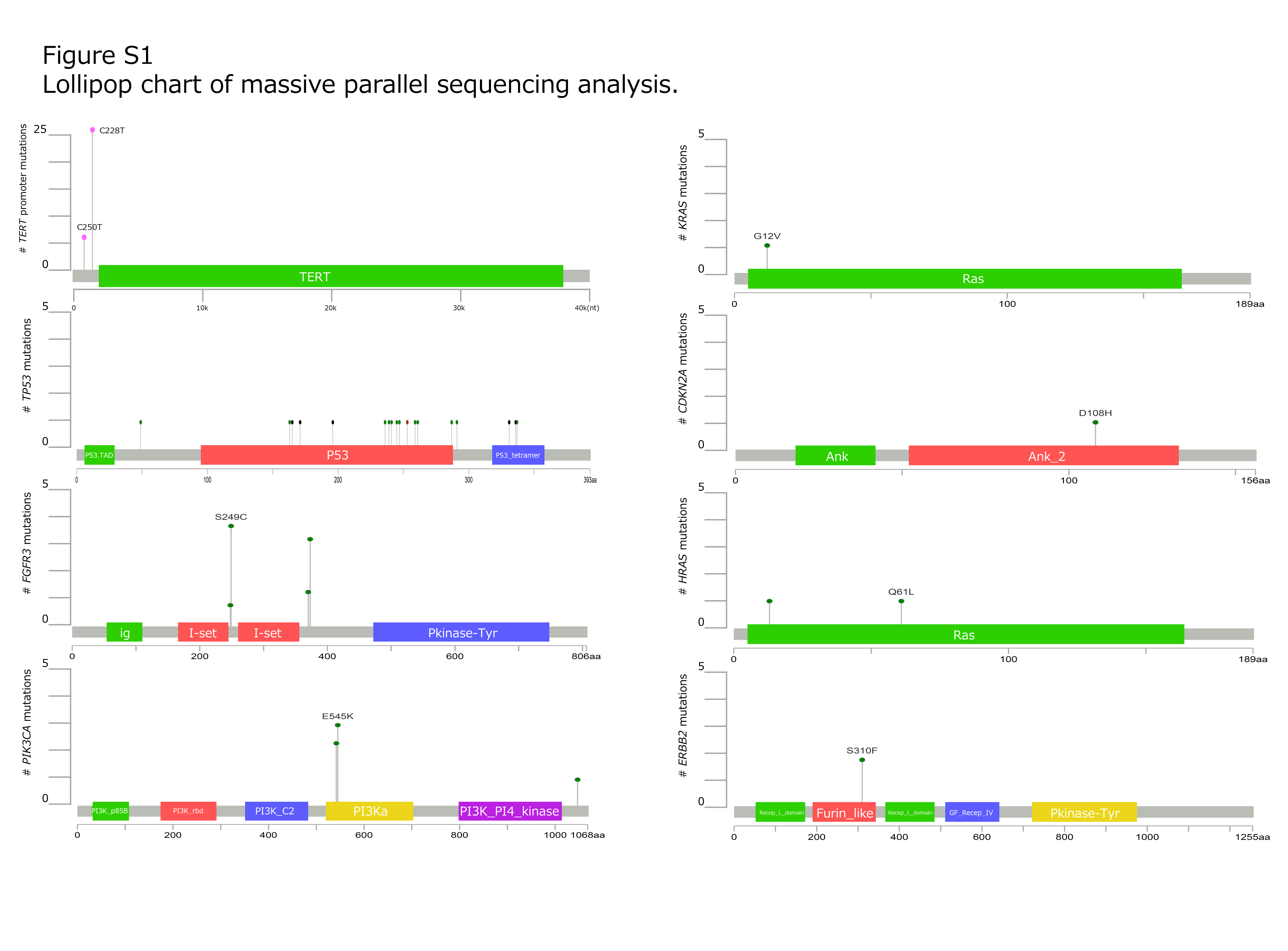

Supplement: Supplementary file 3 [file Image_1.TIF]
